# A Novel Method and Mechanism for Micro-Sphere Singularization

**DOI:** 10.3390/mi8090273

**Published:** 2017-09-08

**Authors:** Gianmauro Fontana, Serena Ruggeri, Luca Altissimo, Giovanni Legnani, Irene Fassi

**Affiliations:** 1 Institute of Industrial Technologies and Automation, Consiglio Nazionale delle Ricerche, Via A. Corti, 12, Milan 20133, Italy; serena.ruggeri@itia.cnr.it (S.R.); luca.altissimo@mail.polimi.it (L.A.); irene.fassi@itia.cnr.it (I.F.); 2 Department of Mechanical and Industrial Engineering, University of Brescia, Via Branze, 38, Brescia 25123, Italy; giovanni.legnani@unibs.it

**Keywords:** mechanism design, singularization, micro-sphere, sorting and feeding, micro-scaled device, micro-assembly

## Abstract

The paper presents an innovative mechanism for the singularization of micro-spheres, which can be effectively employed in a diverse range of robotized applications in micro-electronics and micro-mechanics. Many miniaturized devices are currently being developed and consist of different micro-components to be precisely assembled. The demanding product and process requirements can be met by automating the assembly phases, which include sorting and feeding the micro-components. Therefore, accurate, high-throughput, and modular mechanisms and tools able to supply a number of micro-components, or even a single element for the subsequent operations, play a significant role. In this context, this work focused on the development of a novel strategy for separating a single component from an unstructured stock of identical parts, in particular of micro-spheres with diameters of 0.2–1 mm. Suitable expedients were considered to overcome the adhesive effects that can become significant at the micro-scale due to the very small size and low mass of the micro-spheres. The paper describes the operating principle and the actuation strategies of the mechanism. The design and the development of a prototype for singularizing micro-spheres with a diameter of 0.6 mm are thoroughly discussed. Finally, the results of experimental singularization tests demonstrate the method effectiveness and the mechanism performance.

## 1. Introduction

The assembly of different micro-components to obtain a micro-product is usually the bottleneck and the most expensive phase in the whole manufacturing process due to the multiple difficulties in handling such small parts [1]. For this reason, the automation of the assembly process is fundamental to meet the manufacturing requirements in terms of performance and costs. In particular, the manufacturers need accurate, high-throughput, and modular mechanisms and tools able to pick, orient, move, and release different types of micro-components to join them [1,2]. They should be able to deal with the issues proper of the micro-domain, including the adhesion forces (e.g., capillary, electrostatic, and Van der Waals forces) that can be predominant, hindering the success of the manipulation task. In particular, undesired gripping can occur due to charging effects or the release can be prevented by sticking effects [3]. Among the assembly devices, sorting and feeding systems are essential for an automated assembly, but also challenging, since they influence the productivity and the automation level of the overall manufacturing plant. For example, these systems can (pre-)select the components compliant to the requirements (e.g., to reject defective parts), gather similar components according to specific family criteria, separate and isolate (i.e., singularize) a component from a stock of identical parts, or simply detach and separate components for a reliable grasping. However, the design and development of these systems become more challenging at the micro-scale. Standard macro-mechanisms are not the best choice to prevent the adhesion forces and to reduce the overall dimensions of the system. On the other hand, the downscaling of macro-mechanisms may be infeasible due to the difficulty in finding suitable technologies, e.g., for miniaturized sensors or actuators.

In this context, the paper proposes a novel mechanism for micro-sphere singularization and delivery that can be exploited in various feeding applications, where the delivery of a single micro-sphere is required. Targeted applications are the positioning of solder balls in the assembly of ball grid array (BGA) packages or of micro-ball bearings. However, the proposed mechanism could be successfully exploited also in other cutting edge research fields, such as scanning super-resolution microscopy, where a single micro-sphere has to be attached to the scanning probe [4,5] to obtain scanning systems that can acquire images of sub-diffraction limited targets (i.e., biological or non-biological structures) in a large area using visible light or biological applications, e.g., automated singularization and delivery of biological samples, e.g., eggs for micro-injection [6]. After a brief overview on sorting and feeding devices and mechanisms proposed in literature, the innovative mechanism is introduced, discussing its operating principle and actuation strategies. The design and the related prototype are then presented. The paper reports the experimental tests on the sorting of micro-spheres with diameters of 0.6 mm that have been executed to validate its effectiveness and performance.

## 2. State of the Art

In the world of assembly, the functions provided by sorting and feeding systems are all primary processes and, at the micro-scale, they greatly influence the quality of the final product. Indeed, the risk of damaging the components and their functionality increases. The current solutions used for macro-components show technical limits at the micro-scale and cannot meet all the demanding requirements. For example, the common feeding vibratory technology is affected by the surface effects due to the continuous impact among the small size components. In this context, different methods and mechanisms can be found in the literature to sort and feed micro-parts and managing the adhesion effects. In the following an overview on significant strategies will be presented, focusing on the working principle, the specific functions, and the related applications.

Different devices were designed for the feeding of identical micro-components which are delivered from an upstream storage to the feeding area, arranging them randomly or in a pattern (e.g., by specific masks or along a line).

A mechanism for the simultaneous alignment and ordering of a fixed quantity of micro-spheres in a defined position was developed in [7]. This mechanism is composed of an array of cavities that exploit an airflow to suck the spheres, supporting the alignment.

In [8] a micro-ball feeder to support the assembly of a watch bearing is presented, exploiting a liquid flow to push the spheres. The device allows the separation and arrangement, in specific holes, of 0.5 mm balls delivered in bulk that are then available for the picking by a capillary gripper. Another example of fluidic sorting is reported in [9], where bulk micro-parts are singularized, exploiting the surface tension. A curved liquid surface (concave or convex) is used to allow the sliding of micro-components toward the lateral reservoir rims. 

In [10] the development of a feeder for mini- and micro-parts is presented. The working principle is based on the use of a stepping electric field, generated by V-shaped electrodes, to attract, orient, locate, and transport the micro-parts. The electrodes are mounted over a vibrating platform, useful to reduce friction and adhesion forces in order to transport the micro-components freely on the working area. A commercial example of linear vibrating feeders is the Asycube50 (Asyril SA, Villaz-Saint-Pierre, Switzerland) provided by Asyril SA [11]. It allows the separation, orientation, and moving of a wide range of small components with dimensions from 0.1 to 5 mm, exploiting suitable vibration patterns possibly in combination with structured plates.

Fantoni et al. [12] presented an electrostatic device for parallel sorting of micro-spheres. The sorting was achieved by a matrix of electrode tips to generate an electrostatic potential with several minima where each component is trapped. The micro-parts to be sorted lay on a platform which, during the action of the electric field, starts to vibrate to reduce friction and adhesion.

A method to improve the flexibility in sorting micro-components is the use of a manipulator working in conjunction with a vision sensing. In this case, a vision system detects the position and the orientation (pose) of randomly-scattered micro-parts, and sends the pose information to the manipulator that can pick and finally sort them as required. If the manipulator is able to handle many components simultaneously, the sorting process is sped up [13]. An example of robotized sorting is provided in [14], which presents an automated separation and palletizing of micro-components with a robotic cell employing three cooperating mobile micro-robots. A microscope inspects and classifies the components that are then selected, separated, picked up, and moved to a target pallet. In order to overcome the surface forces between the gripper and the micro-parts, and to enhance the accuracy when placing them, a tip mounted on one of the micro-robots was used.

When the separation of a single component from a stock is necessary, a singularization mechanism has to be provided. For example, in ball bearing manufacturing, some solutions based on mechanical traps have been proposed for the singularization of greased spheres. In [15] a mechanism exploiting a groove and a cone-shaped portion (funnel) is presented.

In the electronics industry, where the component characteristics and the required throughput make the previously-described solutions unfeasible, other mechanisms have been conceived to singularize solder balls and are often adopted to place them in BGA (re-)manufacturing. The working principle can exploit mechanical traps (e.g., holes on rotating disks) [16,17,18], capillaries [19,20], gas flow in chambers [21], and vibration [22]. To cope with the sticking effects occurring among the micro-components and the surrounding surfaces, most of the cited devices integrate systems to support the separation, such as positive pressure, vibration, and energy transfer devices (i.e., mechanical pins). Different solder placement techniques have been used, such as the wafer level solder sphere transfer process (WLSST) [23] where a set of solder balls are simultaneously picked up by a patterned vacuum tooling and transferred on the wafer substrate.

However, these methods present some limitations. For example, some solutions operate in liquid environments and, therefore, cannot be used in electronics. In some cases, the performance of the mechanism is inadequate for industrial applications, providing low throughput. Some devices present complex and rigid structures, while modular and lightweight solutions with simple driving systems and easily mountable on a robotic structure should be preferred. Therefore, the availability of innovative and low-cost singularization solutions would benefit the sorting and feeding processes for the micro-product assembly.

## 3. The Innovative Singularization Mechanism

Micro-spheres are commonly provided as bulk material arranged in unordered batches. The separation of single components is complicated by the above-mentioned adhesive effects. The innovative mechanism described in this paper allows the sorting of a single solder micro-sphere from an unordered stock, to isolate it from the others and deliver it in a specific position for the subsequent phase with high throughput. This mechanism was designed to process spheres with diameters ranging from 0.2 to 1 mm, however, the concept can be extended to a wider range. 

A prototype of the mechanism was realized to experimentally validate its effectiveness and to assess its performance.

### 3.1. Operating Principle of the Singularization Mechanism

**Figure 1:**
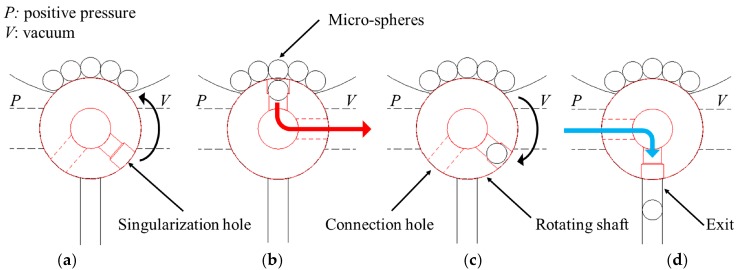
Operating principle of the innovative singularization mechanism (cross-section; dashed lines indicate the holes on a back plane): (**a**) top positioning phase; (**b**) isolation phase; (**c**) bottom positioning phase; and (**d**) exit phase.

**Figure 2:**
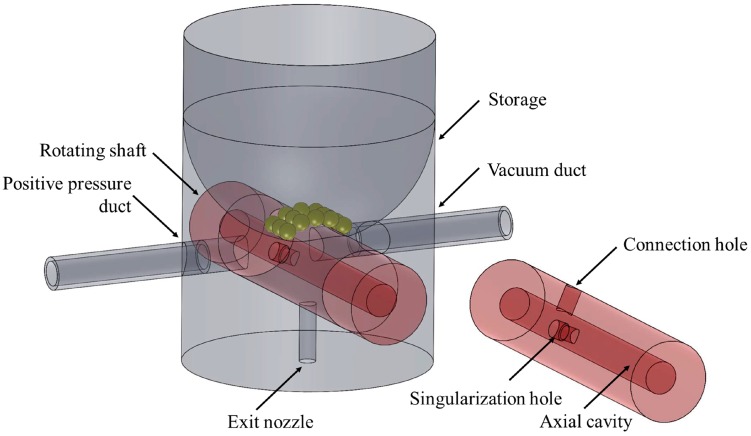
The innovative singularization mechanism.

The conceived principle for the singularization of a micro-sphere from a messy storage containing multiple micro-spheres exploits the rotation of a shaft in the body of the storage (Figure 1 and Figure 2). A through hole in the storage connects it with an exit nozzle on its bottom part. The shaft can rotate inside a specific housing hole in the storage body and it has a radial hole (called the singularization hole) designed to house only one micro-sphere. The rotation axis of the shaft is orthogonal to the axis of the through hole in the storage, therefore the singularization hole can align with the through hole in the storage in two different positions as shown in Figure 1a,d. The inside of the storage, where the micro-spheres are stored, consists of a cavity whose bottom is concave in order to lead the micro-spheres toward the singularization hole on the shaft.

As mentioned above, due to their small dimension, the micro-spheres can stick to each other or onto the storage surfaces and their weight may not suffice to lead and drop a micro-sphere inside the singularization hole; on the other hand, the micro-sphere can stick to the inner surfaces of the singularization hole, causing a delivery failure. For these reasons, the mechanism was equipped with two auxiliary systems to force (i) the sorting of a single sphere from the storage to the singularization hole and (ii) its ejection from the singularization hole.

The negative pressure difference between the shaft and the surrounding environment sucks a micro-sphere inside the singularization hole; oppositely, a positive pressure expulses a micro-sphere from the singularization hole. These pressure differences were provided by suitable air ducts in the mechanism. An axial cavity inside the shaft was then created, as well as a radial hole (the connection hole) connecting to two air sources: a vacuum generator and an air compressor. The connection hole axis is orthogonal to the singularization hole and axially offset from it.

The operating principle of the singularization process is composed by the following phases:

1. Rotation of the shaft to align the singularization hole with the upper part of the through hole inside the micro-spheres storage (top positioning phase);
2. Drop of a micro-sphere into the singularization hole (isolation phase);
3. Rotation of 180° of the shaft to align the singularization hole with the exit nozzle (bottom positioning phase); and
4. Exit of the micro-sphere held into the singularization hole through the exit nozzle (exit phase).

It is worth noting that, as shown in Figure 1, the rotating shaft basically acts as a switching valve that enables the airflow selectively to one of the pressure sources during the micro-sphere isolation and exit phases, while inhibiting the airflow in the other phases. Figure 2 shows the design of the conceived singularization mechanism.

### 3.2. Actuation Strategies for the Singularization Mechanism

The rotation of the axis can be actuated adopting two different strategies: the former considers providing a sequence of 180° rotations in one direction (continuous motion); the latter periodically performs rotations of 180° in opposite directions (intermittent motion).

The first approach can be realized by a continuous motion to achieve a high singularization speed. This strategy requires a precise angular position of the shaft to identify the singularization instant precisely. Indeed, the singularization time feedback is fundamental both to precisely control the number of micro-spheres to singularize and to synchronize the singularization process with other devices, e.g., a positioning manipulator. This strategy can be implemented adopting a rotating actuator equipped with an angular position transducer (e.g., an encoder or a photoelectric sensor) to perform a closed-loop position control, then enabling the synchronization with other devices.

The second approach just requires the shaft rotation to be limited within two positions, corresponding to the isolation and exit positions. Therefore, it is possible to simplify the control of the actuator providing two mechanical stops and driving it to assure the execution of the required rotation without the need of an angular position feedback. In this way, the required singularization throughput can be obtained knowing the dynamics of the system, therefore synchronizing the movements for the isolation and exit. This strategy can be achieved using a rotating or linear actuator controlled in open loop through the two mechanical stops. Since the mechanism already exploits compressed air to operate, a cheap pneumatic linear actuator can be adopted in combination with a mechanical transmission to convert the linear motion to a rotation (e.g., with a rack and pinion).

Both control solutions require a homing (or presetting) phase to identify the position of the motor (then the position of the singularization hole) with respect to the isolation position every time the mechanism is switched on. For the continuous mode, the homing phase should exploit the information of a sensor that can be the same used for the position control in case of an absolute encoder, or another sensor (e.g., a photoelectric sensor) mounted at the isolation/exit position. For the intermittent motion, the homing phase can be achieved by simply rotating the motor (then the shaft) up to the mechanical stop corresponding to the isolation position.

### 3.3. Prototype of the Singularization Mechanism

A prototype of the mechanism was realized in order to validate the effectiveness and the performance of the singularization mechanism. Micro-spheres with diameters of 0.6 mm were considered for the first tests. The mechanism was made of conductive material (metal) to discharge the residual electrostatic charges by grounding and to reduce the adhesive forces. It is mainly composed of two mechanical parts: the rotating shaft and the storage.

**Figure 3:**
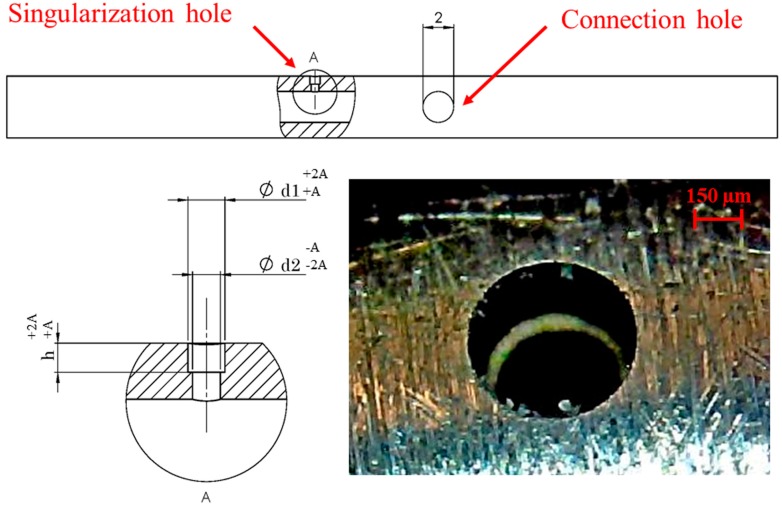
Rotating shaft: characteristic dimensions and detailed view of the manufactured singularization hole.

Firstly, the rotating shaft, which is the main part of the mechanism, was designed. To assure a precise fit with the corresponding housing hole in the storage, the dimensional tolerance on its external diameter was set to an ISO h6 tolerance. Moreover, the shaft was made of stainless steel (EN 1.4301/AISI 304) and manufactured to reduce the sliding friction. The dimensions of the shaft and of the connection hole are the same for all the micro-sphere diameters. Differently, the dimensions of the singularization hole of the shaft have to be designed precisely according to the dimension of the specific micro-sphere in order to house one, and only one, micro-sphere at a time, avoiding its interlocking. Moreover, it has to allow the airflow to support the micro-sphere isolation and exit phases, avoiding the fall of the micro-sphere in the cavity of the shaft. For these reasons, the three dimensions of the singularization hole (that is, the counterbored through-hole shown in Figure 3) were designed according to the nominal dimension of the specific micro-sphere, its manufacturing tolerance, and the desired allowance (which represents the fundamental dimensional deviation, which was set to 15% of the sphere nominal diameter *d_s_* to meet the requirements described above). In particular, the counterbore height and diameter depend on the maximum diameter of the micro-sphere, while the through-hole diameter is related to the minimum sphere diameter. Table 1 reports the design of the singularization hole according to the different dimensions and tolerances of the micro-spheres available for testing [24], and the rounded allowance values. However, the desired allowance on the through-hole can be neglected by reducing its diameter and designing it considering the required forces to support the isolation and exit phases. The singularization hole was then manufactured by a micro-Electrical Discharge Machining (EDM) process with a SX-200-HPM (Sarix SA, Sant’Antonino, Switzerland) machine (see Figure 3).

The storage, common for all the diameters of the micro-spheres, was manufactured to be modular for operating with all the shaft formats. In particular, it has to contain the desired number of micro-spheres to singularize inside a cylindrical cavity whose lower part is semi-spherical and has a bottom opening to lead the micro-spheres toward the singularization hole of the shaft. Moreover, the storage should allow the rotation of the shaft inside its body without undesired clearance that would cause air leakage and consequent stress inside the mechanism due to pressure fluctuations. For this reason, as said above, a precise clearance fit between the shaft and the corresponding housing hole in the storage is essential. For the current prototype, an ISO H7-h6 fit was considered suitable. The storage was then made of brass and the hole was manufactured to minimize the friction during the shaft rotation and the driving torque, consequently. In addition, the storage should allow the exit of the singularized micro-sphere. For this reason, a through hole was realized to connect the inside of the storage to the output nozzle. This hole was designed to allow the transfer of the micro-spheres regardless of their dimensions. Its diameter derives from the maximum diameter of the biggest micro-sphere to sort, i.e., by adding the largest micro-sphere diameter (0.76 mm), the positive tolerance on its diameter, and the desired allowance. Finally, the storage has to allow the airflow by the vacuum generator and the compressor inside the cavity of the shaft through the connection hole. The two air ducts were realized radially to the shaft housing hole according to the angular positions of the connection hole in the isolation/exit phases. The diameters of the ducts are the same as that of the connection hole. The air sources were then connected to the air ducts by quick-connect fittings and operated continuously during the process.

A conical hollow needle was mounted under the exit nozzle to facilitate the exit path and precise positioning of the spheres. Its tip internal diameter *d_n_* has to be designed considering the specific micro-sphere diameter *d_s_* to make it fall within a specified limited area. More in detail, *d_n_* ≥ *d_s_ + t + f_d_*, where *t* is the positive manufacturing tolerance on *d_s_* and *f_d_* is the fundamental dimensional deviation to avoid the sphere blocking at the exit. Adopting commercial needles available in a range of internal diameters *d_i_*, the needle to pursue the highest precision will have d_i_^*^ = min (d_i_ ≥ d_n_).

For the current prototype, the needle with an internal diameter of 0.838 mm was selected among the range of commercially-available needles for liquid deposition (TT needles, Techcon Systems, Cypress, CA, USA [25]).

The conceived mechanism is modular since, in order to singularize a different sphere diameter, it is only necessary to change the rotating shaft and the needle.

**Figure 4:**
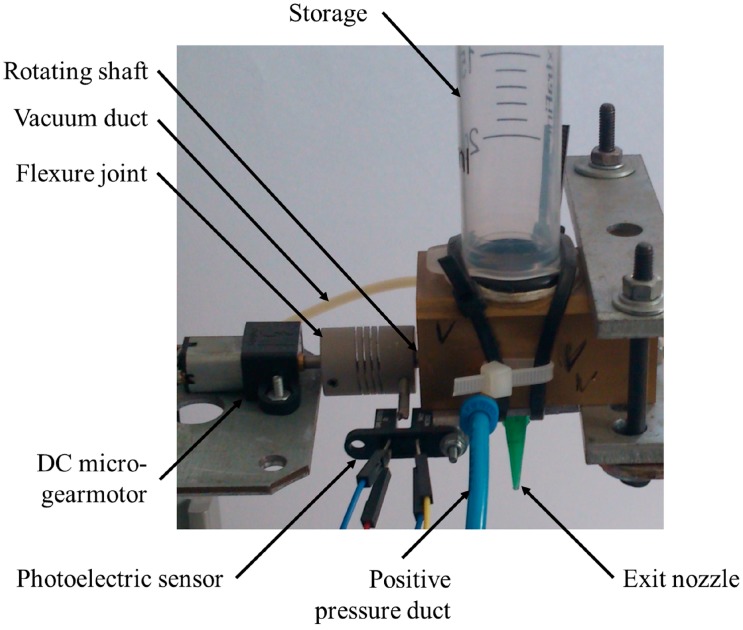
Developed prototype of the singularization mechanism for micro-spheres with diameters of 0.6 mm.

For the development of the prototype, the continuous motion with a rotating actuator was adopted because it allows achieving very high performance in terms of singularization throughput, not reachable with the intermittent actuation. Indeed, keeping the actuator performance constant (i.e., velocity and acceleration) and the number of the singularization cycles, the intermittent rotation is characterized by continuous transients of acceleration and deceleration that increase the actuation time with respect to the continuous rotation where the transients of acceleration and deceleration only occur at the beginning and at the end, respectively. Therefore, the prototype was equipped by a rotating DC micro-gearmotor (HP 6V 30:1, Pololu Robotics and Electronics, Las Vegas, NV, USA) chosen to overcome the static and dynamic friction between the shaft and the housing hole (that represents the motor load). The system dynamics were identified to perform an open-loop control of the actuator. An open-loop control was sufficient at this stage of the research devoted to the identification of the maximum performance of the systems in terms of sphere throughput. A closed-loop solution will be mandatory in the final version of the system where synchronization with other devices is fundamental. The motor was fixed axially to the shaft by means of a flexure joint. Moreover, a fork-type through-beam photoelectric sensor (OPB865T51, TT Electronics, Woking, UK) was mounted to identify the instant when the singularization hole is in the exit position during the operation and to perform the homing phase during the startup. Moreover, this sensor provides the measure of the actual time for a single shaft rotation. Figure 4 shows the developed prototype of the singularization mechanism for micro-spheres with diameters of 0.6 mm.

## 4. Experimental Tests on the Singularization Mechanism

Once the prototype of the mechanical part of the singularization device for micro-spheres with diameters of 0.6 mm was complete, a full experimental setup was developed to test its effectiveness and performance in terms of reliability and throughput. The experimental setup should control the mechanism, measure the number of singularized micro-spheres over the set number of cycles, and should allow for varying the operating parameters of the mechanism to characterize it under different working conditions.

**Figure 5:**
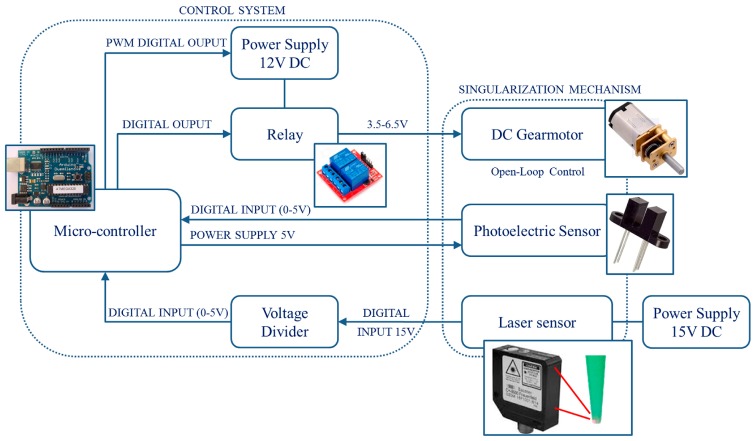
Block diagram of the experimental setup.

The Arduino UNO micro-controller (BCMI Industries SA, Chiasso, Switzerland) was chosen for the control of the rotation of the motor attached to the mechanism shaft and for the monitoring of the mechanism during operation. The DC rotating motor was driven in open-loop control by the micro-controller through a relay. The input voltage could be set by software within the admissible range (3.5–6.5 V) by means of a stabilized power supply to vary the rotation speed and, therefore, the singularization throughput. Indeed, adopting the continuous motion, the singularization throughput corresponds to the shaft speed (in revolutions per second) that depends on the motor speed. Moreover, the micro-controller collects the data from the photoelectric sensor, detecting the instant when the singularization hole passes by the exit position, then measuring the actual time for a single shaft rotation. Finally, the micro-controller was interfaced to an optical sensor able to determine the instant when the micro-spheres exited the mechanism. A laser sensor for contrast detection was selected (OZDM 16P1001/S14, Baumer, Frauenfeld, Switzerland) and calibrated to detect the exit of the singularized micro-spheres from the needle nozzle. Both of the signals coming from the two sensors were treated as interrupt signals, in order to monitor them at the Arduino clock speed (16 MHz, ~63 ns) without any data loss. The system was equipped with a push button to start the test. Figure 5 shows a block diagram of the developed setup.

After the setup of the experimental equipment, the tests were planned and the control algorithms developed. Since the tests were finalized to identify the number of singularized spheres over the set number of singularization cycles depending on the rotation speed, each test was divided into the phases described in the following.

### 4.1. Selection of the Operating Parameters

The parameter that affects the throughput of the system is the motor speed, then the shaft speed. Such a speed can be varied by acting on the input voltage since the achieved torque is always enough to overcome the friction between the shaft and the housing hole. It has to be noticed that increasing the rotation speed, the number of singularization cycles increases, then the motor driving time was varied according to its speed to keep the number of singularization cycles constant in each test. This can be done knowing the dynamics of the system. Therefore, each test was executed, increasing the motor input voltage within the admissible range in order to obtain increasing speeds, while decreasing the motor driving time to obtain the same number of cycles. Other parameters do not directly affect the throughput, but influence the effectiveness and the reliability of the singularization. These parameters are the positive and negative pressure values supporting the isolation and exit phases. In the current tests, these parameters were kept constant for the assessment of the reliability of the mechanism. The values were identified driving the mechanism manually and checking that, at a low throughput, the isolation and exit of the micro-sphere were always successful. The chosen value for the negative pressure was equal to −5 kPa, while the positive pressure was set to +80 kPa.

### 4.2. Test Execution

To validate the singularization mechanism, five tests at five different speeds were performed. For each test, the experimental procedure was developed as follows: Once the start signal generated by pushing the start button is received, the micro-controller activates, for a specified actuation time, the relay that enables the power supply with the chosen input voltage; in parallel, the micro-controller receives the signals from the photoelectric sensor and the laser sensor to determine the instants at which the shaft completes a rotation (passing by the exit position) and the micro-sphere is expelled. The micro-spheres were collected in a container and, at this step, their positioning precision was not assessed. Once the motor driving time, corresponding to the specific test, is expired, the micro-controller deactivates the relay to stop the motor (then the shaft rotation) in order to achieve the desired number of cycles for each test. In this work, 30 cycles were considered suitable for the preliminary assessment of the mechanism reliability.

### 4.3. Assessment of the Performance of the Mechanism

After the test execution, the collected data, coming by the two sensors, were analyzed to assess the mechanism performance. In particular, the reliability was defined as the number of expelled micro-spheres over the number of expected micro-spheres (that is, the number of set cycles); the throughput was then calculated as the number of expected micro-spheres over the motor driving time for the specific test. In this case, the throughput will correspond to the rotation average speed of the shaft in revolutions per second.

**Figure 6:**
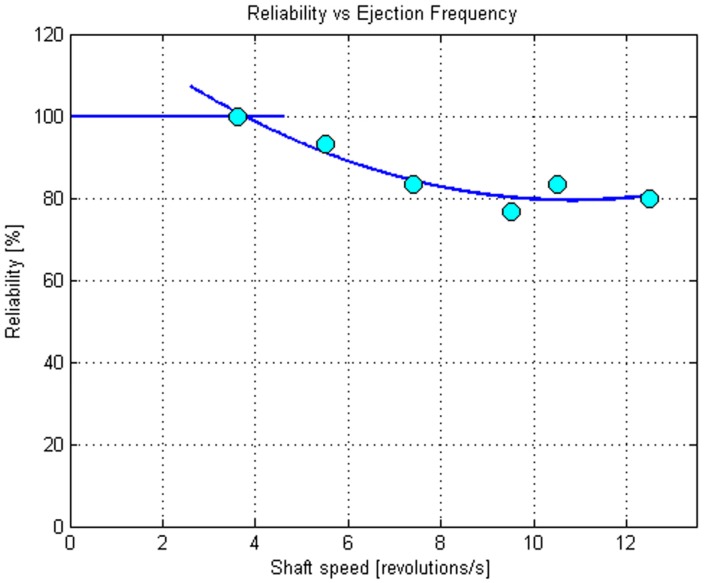
Graphical representation of the reliability trend for the singularization tests with 0.6 mm micro-spheres.

**Figure 7:**
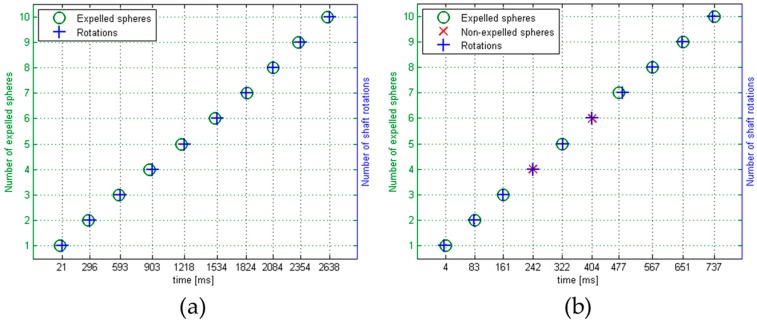
Plots of two reliability tests at different actuator speeds: (**a**) the test at 3.6 revolutions/s with 10 over 10 expelled micro-spheres; and (**b**) the test at 12.5 revolutions/s with eight over 10 expelled micro-spheres.

This analysis was repeated for different tests varying the above-cited parameters. Table 2 summarizes the achieved results and shows a decrease in reliability as the motor speed increases, clearly visible in Figure 6. This data states that, at present, the maximum throughput is a little less than four micro-spheres per second. In Figure 7 two examples of reliability tests in continuous mode at different speeds are displayed. On the X-axis the time is reported, while the Y-axis shows the number of shaft rotations and the number of expelled spheres. A circular marker (O) indicates the expelled micro-spheres, while a cross (X) indicates if a micro-sphere was not expelled during that shaft rotation. The missed expulsion was due to the blocking of the micro-sphere in the singularization hole. This can be due to two causes; the former is a micro-sphere diameter slightly larger than expected, so that the counterbore on the singularization hole is too small to house the micro-sphere properly. To avoid this possible issue, the counterbore should have a larger diameter, that is, a larger allowance value. On the other hand, a failed micro-sphere exit can occur if the positive pressure is not enough to overcome the adhesion effect between the micro-sphere and the singularization hole. The missed expulsion could be also due to the unsuccessful isolation of a micro-sphere. In this case, it means that the micro-sphere was not falling into the singularization hole on the shaft. This can occur, for example, if more micro-spheres simultaneously approach the bottom opening of the concave part of the storage, getting blocked and occluding the passage to a single micro-sphere. Moreover, the negative pressure that sucks the micro-sphere in could be insufficient. For this reason, a deeper study on the influence of the pressure values could benefit the design of the auxiliary systems. In addition, the micro-sphere could not exit from the nozzle due to a misalignment between the singularization hole and the storage opening connecting to the exit nozzle, caused for example by axial vibrations of the shaft at high speed. Therefore, the design and mounting of the mechanism should be optimized to avoid these undesired vibrations.

## 5. Conclusions

The paper proposes a novel method and mechanism for micro-sphere singularization and delivery. The mechanism was designed in a modular way to be able to singularize micro-spheres with dimensions in the range 0.2–1 mm. It could be integrated into complex devices to perform specific micro-sphere sorting as part of different tasks and applications. A possible application is the (re-)balling of BGA packages. Indeed, the mechanism can provide, when required, a solder micro-sphere to realize the electrical connections. Adopting the conceived singularization mechanism, the picking phase is not needed, and a very high process throughput can be obtained. In this case, the exit nozzle should be equipped with a precise deposition needle that has to be designed with very tight manufacturing tolerances to allow an accurate placement of the singularized spheres on the pads of the BGA substrate.

A prototype to singularize micro-spheres with a diameter of 0.6 mm was realized and tested to validate its effectiveness at different speeds. The experimental tests showed the interesting capabilities of the conceived method, proving excellent reliability at low throughput. At the present stage, experimental results on 30 cycles showed a throughput of about four micro-spheres per second with a reliability of 100%.

On the contrary, when the throughput increased, its effectiveness worsened. This result could be improved by an optimized design and manufacturing. Indeed, further research will consider the development of a multi-physical model for the simulation of the singularization process to highlight the critical issues for a focused improvement.

## Figures and Tables

**Table 1:** Singularization hole main dimensions according to the different manufacturing tolerances of the micro-spheres (solder balls: alloy: 63Sn–37Pb series eutectic composition).

Diameter *d_s_* (mm)	Tolerance (mm)	Allowance *A* (mm)	*d*1 (mm)	*d*2 (mm)	*h* (mm)
0.760	±0.020	0.110	0.780	0.740	0.780
0.650		0.100	0.670	0.630	0.670
0.600		0.090	0.620	0.580	0.620
0.550	±0.015	0.080	0.565	0.535	0.565
0.500		0.080	0.515	0.485	0.515
0.450		0.070	0.465	0.435	0.465
0.400		0.060	0.415	0.385	0.415
0.350	±0.010	0.050	0.360	0.340	0.360
0.300		0.050	0.310	0.290	0.310
0.250		0.040	0.260	0.240	0.260

**Table 2:** Results of the singularization tests with 0.6 mm micro-spheres.

Tests	1	2	3	4	5	6
Shaft speed (revolutions/s)	3.6	5.5	7.4	9.5	10.5	12.5
Positive pressure (kPa)	80	80	80	80	80	80
Vacuum (kPa)	−5	−5	−5	−5	−5	−5
Set cycles	30	30	30	30	30	30
Singularized spheres	30	28	25	23	25	24
Reliability (%)	100	93.3	83.3	76.6	83.3	80
Expected Throughput (spheres/s)	3.6	5.5	7.4	9.5	10.5	12.5

## References

[1] Porta M., Fantoni G., Lambert P. (2010). An integrated and compact device for microassembly exploiting electrostatic sorting and capillary grasping. CIRP J. Manuf. Sci. Technol..

[2] Fontana G., Ruggeri S., Fassi I., Legnani G. (2014). A mini work-cell for handling and assembling microcomponents. Assem. Autom. J..

[3] Pagano C., Fassi I., Fassi I., Shipley D. (2017). Introduction to miniaturization, Chapter 1. Micro-Manufacturing Technologies and Their Applications: A Theoretical and Practical Guide.

[4] Yan Y.Z., Li L., Feng C., Guo W., Lee S., Hong M. (2014). Microsphere-coupled scanning laser confocal nanoscope for sub-diffraction-limited imaging at 25 nm lateral resolution in the visible spectrum. ACS Nano.

[5] Wang F., Liu L., Yu H., Wen Y., Yu P., Liu Z., Wang Y., Li W.J. (2016). Scanning superlens microscopy for non-invasive large field-of-view visible light nanoscale imaging. Nat. Commun..

[6] Truong H.H., De Sonneville J., Ghotra V.P.S., Xiong J., Price L., Hogendoorn P.C.W., Spaink H.H., Van De Water B., Danen E.H.J. (2012). Automated microinjection of cell-polymer suspensions in 3D ECM scaffolds for high-throughput quantitative cancer invasion screens. Biomaterials.

[7] Brecher C., Weinzierl M., Ratchev S., Koelemeijer S. (2008). Manufacturing of devices for the parallel precision alignment of multiple micro components. Micro-Assembly Technologies and Applications, Proceedings of the IFIP—International Federation for Information Processing; Chamonix, France, 10-13 February 2008.

[8] Lenders C., Valsamis J.B., Desaedeleer M., Delchambre A., Lambert P., Ratchev S., Koelemeijer S. (2008). Assembly of a micro ball-bearing using a capillary gripper and a microcomponent feeder. Micro-Assembly Technologies and Applications, Proceedings of the IFIP—International Federation for Information Processing; Chamonix, France, 10-13 February 2008.

[9] Burgard M., Othman N., Mai U., Schlenker D., Verl A., Ratchev S. (2014). Feeding of small components using the surface tension of fluids. Precision Assembly Technologies and Systems, Proceedings of the IFIP Advances in Information and Communication Technology.

[10] Fantoni G., Santochi M. (2005). A modular contactless feeder for microparts. CIRP Ann..

[11] Asycube 50: Flexible Vibrating Feeder for Parts from 0.1 to 5 mm by Asyril SA. http://www.asyril.com/en/products/asycube-flexible-feeders.html.

[12] Fantoni G., Porta M., Santochi M. (2007). An electrostatic sorting device for microparts. CIRP Ann..

[13] Naito K., Kanisawa K., Fujimori Y. (2005). Ball Mounting Device. JP Patent.

[14] Jasper D., Diederichs C., Stolle C., Fatikow S. Automated robot-based separation and palletizing of microcomponents. Proceedings of the 2011 IEEE International Symposium on Assembly and Manufacturing (ISAM).

[15] Manfred B. (2012). Device for Separating Spherical Objects, has Outlet Opening for Separated Spherical Objects, which is Provided in Transition Area between Cone-Shaped Bottom Portion and inner Wall of Cylindrical Receiving Container. German Patent.

[16] Oppert T., Titerle L., Zakel E., Azdasht G., Teutsch T. Placement and reflow of solder balls for FC, BGA, wafer-level-CSP, optoelectronic components and MEMS by using a new solder jetting method. Proceedings of the International Symposium on Microelectronics.

[17] Azdasht G. (2015). Apparatus for the Individual Application of Deposits of Connecting Material. International Patent.

[18] Zhang R., Yan X., Tang H. (2014). Welding device for ball type welding flux. Chinese Patent.

[19] Azdasht G. (1997). Process and Apparatus for Producing a Bonded Metal Coating. U.S. Patent.

[20] Eldring J., Jung E., Zakel E. (1996). Process and Device for Applying Bonding Material to a Substrate Connection Surface. International Patent.

[21] Ito C.T. (2004). Solder Ball Dispenser. U.S. Patent.

[22] Nakano H., Kawakami N., Amano Y., Takasu K., Hatano K. (1995). Method for separating small solder. JP Patent.

[23] Oppert T., Teutsch T., Azdasht G., Zakel E. Micro ball bumping packaging for wafer level & 3-d solder sphere transfer and solder jetting. Proceedings of the 35th IEEE/CPMT International Electronics Manufacturing Technology Conference (IEMT).

[24] JOVY SYSTEMS^®^ BGA Sphere Balls Diameter Details by Jovy Systems. http://www.jovy-systems.com/en/products/chemicals-a-consumables/sphere-solder-balls.html.

[25] Dispensing Products—Data Sheets (Dispensing Needles) by Techcon Systems. http://www.techconsystems.com/en/dispensing-tips/taper-tips/.

